# Appropriateness of gastrointestinal prophylaxis use during hospitalization in patients with acute myocardial infarction: Analysis from the China Acute Myocardial Infarction Registry

**DOI:** 10.1002/clc.23449

**Published:** 2020-11-19

**Authors:** Wence Shi, Lin Ni, Jingang Yang, Xiaoxue Fan, Mei Yu, Hongmei Yang, Mengyue Yu, Yuejin Yang, China Acute Myocardial Infarction (CAMI) Registry study group

**Affiliations:** ^1^ Department of Cardiology National Center of Cardiovascular Disease, Chinese Academy of Medical Sciences and Peking Union Medical College, Fuwai Hospital Beijing China; ^2^ Medical Research and Biometrics Center, State Key Laboratory of Cardiovascular Disease Fuwai Hospital, National Center for Cardiovascular Diseases, Chinese Academy of Medical Sciences and Peking Union Medical College Beijing China; ^3^ Department of Cardiology Langfang People's Hospital Langfang Hebei Province China; ^4^ Department of Cardiology First Hospital of Qinhuangdao Qinhuangdao China

**Keywords:** acute myocardial infarction, appropriateness, co‐medication, outcomes, proton pump inhibitors

## Abstract

**Background:**

The current status of gastrointestinal prophylaxis (GIP) usage and its effects on hospitalized acute myocardial infarction (AMI) patients is not clear. We investigate the appropriateness of GIP usage and its relationship with clinical events in China.

**Hypothesis:**

Appropriate use of GIP is not associated with increased adverse outcomes.

**Methods:**

From January 2013 to September 2014, a total of 24 001 consecutive patients from 108 hospitals with AMI in China Acute Myocardial Infarction (CAMI) registry were analyzed. The appropriateness of GIP was evaluated using the current American College of Cardiology Foundation/American Heart Association (ACCF/AHA) and European Society of Cardiology (ESC) guidelines. The primary endpoint was in‐hospital gastrointestinal bleeding (GIB), while the secondary endpoints were in‐hospital and 2‐year follow‐up net adverse cardiovascular and cerebrovascular events (NACCE). Multivariate logistic regression analysis and Cox proportional hazard models were used to assess the effect of appropriate GIP.

**Results:**

There were 16 413 (68.38%) AMI patients co‐medicated with GIP. Among 108 involved hospitals, only 35 (32.4%) hospitals prescribed more than 50% appropriate GIP. Totally, 59.7% (14 340) AMI patients received inappropriate GIP. Inappropriate GIP use was independently associated with use of GPIIb/IIIa receptor inhibitor and primary percutaneous coronary intervention (PCI). Moreover, appropriate GIP use was associated with decreased GIB risk (OR: 0.692, 95% CI: 0.507‐0.944, *P* = .0202) during hospitalization, while not with increased in‐hospital and 2‐year follow‐up NACCE.

**Conclusion:**

The use of GIP is prevalent in patients with AMI in China but only 40% of hospitalized patients received appropriate GIP. Appropriate prophylactic therapy was associated with decreased GIB risk during hospitalization.

## 1 INTRODUCTION

Antiplatelet and antithrombotic therapy are cornerstone in the treatment of acute myocardial infarction (AMI).^1^ However, they have been associated with increased risk of major bleeding events, including gastrointestinal bleeding (GIB) and access site bleeding. GIB is related to an increased in‐hospital mortality^2^; the real‐world rates of GIB vary from 1.3% to 3%.^3^, ^4^ The American College of Cardiology Foundation/American Heart Association (ACCF/AHA)^5^ and European Society of Cardiology (ESC)^6^ guidelines recommended that gastrointestinal prophylaxis (GIP), especially proton pump inhibitors (PPIs), should be used in high‐risk patients, such as those with a history of GIB, advanced age, concomitant use of warfarin, steroids, or *Helicobacter pylori* infection.

GIP use rate varies from 23% to 45%^7^, ^8^ as reported in large‐scale, multicenter registry studies, with 30% to 60% GIP use were considered inappropriate.^9^, ^10^ In several cases, the use of PPIs may have a potential negative interference on antiplatelet effect of clopidogrel, because of the competitive inhibition of the CYP 2C19 isoenzyme,^11^, ^12^ with higher rates of adverse clinical events in some studies.^12^ Our aim was to describe the current status of GIP use in AMI patients and assess the effect of appropriate GIP use on clinical outcomes in China.

## 2 METHODS

The China Acute Myocardial Infarction (CAMI) registry is a prospective, nationwide, multicenter observational study of patients with AMI. The registry includes three levels of hospitals (provincial‐, prefectural‐, and county‐level hospitals, representing typical Chinese governmental and administrative models) covering all provinces and municipalities across mainland China. The final inclusion criteria met the third Universal Definition for Myocardial Infarction (2012).^13^ The CAMI registry was registered with ClinicalTrials.gov (NCT01874691) and was approved by the institutional review board of all participating hospitals. All patient data were protected at all times. Detailed descriptions about data management and quality control can be found in the methodological article about CAMI registry published previously.^13^ The data analyzed in our research was from CAMI, which could provide a large‐scale population.

We refer to the definition of appropriate GIP use according to the study of Morneau KM^14^ and latest guideline.^6^ Appropriate GIP in patients undergoing dual antiplatelet therapy (DAPT) was defined as the following: indicated for GIP and received PPIs or no indication and did not receive GIP. Inappropriate prophylaxis was defined as no indication represent yet received GIP or that GIP was indicated, but received incorrect prophylaxis (histamine‐2 receptor antagonist [H2RA]) or no prophylaxis.^6^ (Table S1) GIP indication was defined according to the guideline,^15^ including advanced age (>75), concurrent use of anticoagulants, steroids, or nonsteroidal antiinflammatory drugs (NSAIDs), including aspirin and *H*. *pylori* infection. The primary endpoint was in‐hospital GIB, which was defined as clinically evident GIB (gross hematemesis, heme positive coffee ground emesis, and heme positive melena). The secondary endpoints were GIB and net adverse cardiovascular and cerebrovascular events (NACCE) during a 2‐year follow‐up; NACCE was a composite of all‐cause death, myocardial infarction, stroke, and BARC 3 or 5 bleeding.^16^ Post‐discharge study follow‐up was conducted via centralized telephone interviews by trained personnel at 30 days, 6 months and 2 years. GIP use was identified at study baseline and at each study follow‐up. Patients were excluded if they had changed their initial GIP status (appropriate or inappropriate) during the follow‐up.

**FIGURE 1:**
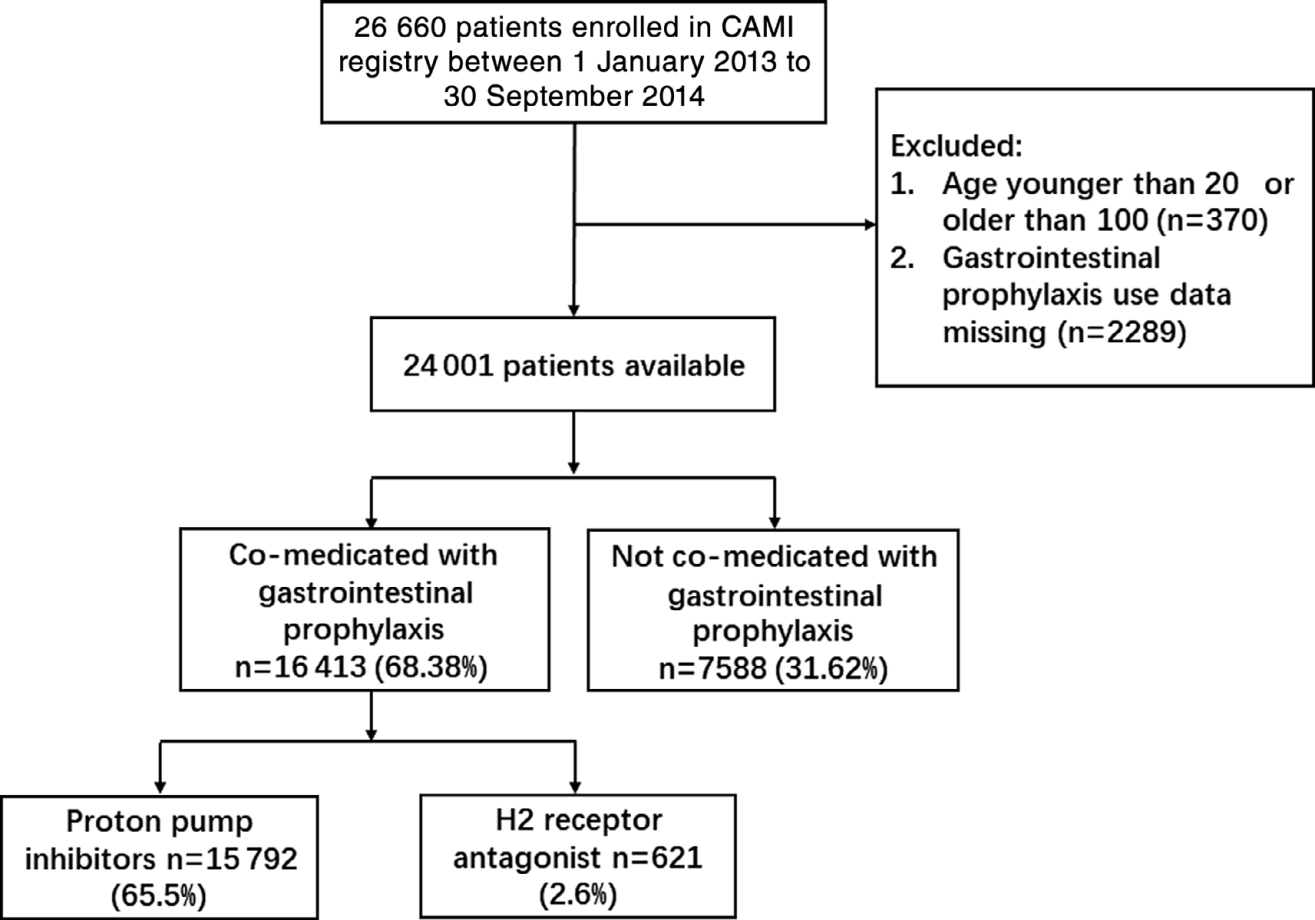
Flow chart for selection of study population

Patients with incorrect age and missing GIP data were excluded. (Figure 1) Continuous variables are expressed as mean SD or median (25th and 75th percentiles), and categorical variables are presented as percentages. Differences in baseline characteristics and outcomes in patients with and without GIP were assessed using the chi‐square test or Fisher's exact test for categorical variables and analysis of variance test or the Wilcoxon rank test for continuous variables. Independent factors associated with inappropriate GIP prescribing were identified using multivariate logistic regression analysis. Multivariate logistic regression analyses were also conducted to evaluate the adjusted effect of appropriate GIP use on GIB. The 2‐year follow‐up NACCE rate was modeled using Cox proportional hazard regression. Clinical characteristics that were imbalanced at a nominal 5% significance level between the two groups treated or not treated with appropriate GIP were identified and included the final adjusted model; these included age, clinical presentation, and medicine therapy. (Detailed variables included were presented below the relevant tables) odds ratio (OR) and hazard ratio (HR) were presented with the 95% confidence intervals (CIs). All statistical analyses were performed using SAS version 9.4, and a two‐tailed *P* < .05 was considered statistically significant. All *P*‐values are to be considered exploratory or hypothesis‐generating in nature.

## 3 RESULTS

### 3.1 Current status of GIP

After excluding 2659 patients, a total of 24 001 consecutive patients from 108 hospitals were analyzed. There were 16 413 (68.38%) AMI patients co‐medicated with GIP, including PPI (15 792, 65.80%) and H2RA (621, 2.59%). Demographic and clinical characteristics are presented in Table 1. Patients treated with GIP were inclined to be female, older, with a higher Killip class on admission and with a history of stroke, peptic ulcer, and GIB. On hospitalization, they were often treated with GPIIb/IIIa receptor inhibitor, heparin/low molecular weight heparin (LMWH), and DAPT. When compared with patients treated with PPIs, those treated with H2RA were likely diagnosed with non‐ST elevated myocardial infarction, and proportion of patients who underwent thrombolysis was higher than patients treated PPIs.

**TABLE 1:** Baseline clinical data in patients with and without GIP

Variables	No GIP (n = 7588)	GIP				*P* value^b^
		Total (n = 16 413)	PPIs (n = 15 792)	H2RA (n = 621)	*P* value^a^	
Demographics						
Age	61.62 ± 13.62	62.65 ± 13.37	62.65 ± 13.37	62.80 ± 13.67	<.0001	<.0001
≥ 75, n (%)	1258 (16.6%)	3112 (19.0%)	2998 (19.0%)	114 (18.4%)	<.0001	<.0001
Female, n (%)	1858 (24.5%)	4207 (25.6%)	4048 (25.6%)	159 (25.6%)	.1634	.0570
Medical history, n (%)						
Hypertension	3647 (48.2%)	8422 (51.4%)	8096 (51.3%)	326 (52.8%)	<.0001	<.0001
Dyslipidemia	467 (6.2%)	1166 (7.1%)	1110 (7.0%)	56 (9.1%)	.0040	.0060
Diabetes mellitus	1340 (17.7%)	3253 (19.9%)	3142 (20.0%)	111 (18.0%)	.0002	.0001
Myocardial infarction	528 (7.0%)	1179 (7.2%)	1136 (7.2%)	43 (7.0%)	.8137	.5374
PCI	339 (4.5%)	785 (4.8%)	761 (4.8%)	24 (3.9%)	.3220	.2845
CABG	32 (0.4%)	65 (0.4%)	63 (0.4%)	2 (0.3%)	.9152	.7699
Congestive heart failure	158 (2.1%)	409 (2.5%)	390 (2.5%)	19 (3.1%)	.0934	.0499
Stroke	590 (7.8%)	1592 (9.7%)	1537 (9.8%)	55 (9.0%)	<.0001	<.0001
Peripheral arterial disease	27 (0.4%)	114 (0.7%)	108 (0.7%)	6 (1.0%)	.0028	.0008
Chronic kidney disease	92 (1.2%)	212 (1.3%)	199 (1.3%)	13 (2.1%)	.2184	.6126
PUD/*Helicobacter pylori*	81 (1.1%)	600 (3.7%)	579 (3.7%)	21 (3.4%)	<.0001	<.0001
GIB	67 (0.9%)	350 (2.1%)	338 (2.1%)	12 (1.9%)	<.0001	<.0001
Malignancy	79 (1.0%)	222 (1.4%)	216 (1.4%)	6 (1.0%)	.0853	.0416
Admission features						
NSTEMI, n (%)	2039 (26.9%)	3937 (24.0%)	3774 (23.9%)	163 (26.2%)	<.0001	<.0001
STEMI, n (%)	5549 (73.1%)	12 476 (76.0%)	12 018 (76.1%)	458 (73.8%)	<.0001	<.0001
Heart rate (beats/min)	78.15 ± 19.06	78.03 ± 18.76	78.02 ± 18.80	78.38 ± 17.74	.7991	.6344
Systolic BP (mm Hg)	129.65 ± 26.14	128.62 ± 25.48	128.63 ± 25.53	128.27 ± 24.26	.0148	.0043
Killip class ≥ II	1643 (21.6%)	4399 (26.9%)	4223 (26.7%)	176 (28.3%)	<.0001	<.0001
Ccr (mL/min·1.73 m^2^) ≤30	409 (5.4%)	662 (4.1%)	634 (4.0%)	28 (4.5%)		<.0001
Hb (g/dL)	13.6 ± 2.2	13.5 ± 2.1	13.5 ± 2.1	13.7 ± 2.3	.0006	.0009
Hct (%)	38.92 ± 14.57	40.39 ± 49.26	40.41 ± 50.09	39.78 ± 14.35	.0442	.0006
LVEF (%)	53.60 ± 10.50	53.39 ± 11.12	53.40 ± 11.12	52.91 ± 11.27	.3200	.2072
CRUSADE score	20.03 ± 15.36	19.99 ± 15.24	19.96 ± 15.23	20.69 ± 15.64	.5013	.8690
In‐hospital medications, n (%)						
Asprin	7303 (96.4%)	15 870 (96.8%)	15 268 (96.8%)	598 (96.5%)	<.0001	<.0001
P2Y12 receptor inhibitor	7186 (95.4%)	15 936 (97.6%)	15 341 (97.6%)	595 (97.2%)	<.0001	<.0001
GPIIb/IIIa receptor inhibitor	1773 (24.5%)	5370 (33.7%)	5251 (34.2%)	119 (20.4%)	<.0001	<.0001
oral anticoagulants	213 (2.9%)	171 (1.1%)	154 (1.0%)	17 (2.8%)	<.0001	<.0001
Heparin/LMWH	6437 (86.7%)	14 824 (92.2%)	14 301 (90.6%)	523 (87.6%)	<.0001	<.0001
DAPT	7014 (92.4%)	15 397 (93.8%)	14 818 (93.8%)	579 (93.2%)	.0004	.0001
Steroids	89 (1.2%)	192 (1.2%)	183 (1.2%)	9 (1.5%)	.0614	<.0001
β‐blockers	5098 (67.3%)	11 748 (71.7%)	11 338 (71.9%)	410 (66.5%)	<.0001	<.0001
ACEI/ARB	4442 (58.6%)	10 035 (61.3%)	9647 (61.3%)	388 (63.3%)	<.0001	<.0001
Treatment, n (%)						
Primary PCI	2673 (35.2%)	6020 (36.7%)	5835 (37.0%)	185 (29.8%)	<.0001	<.0001
Emergent CABG	22 (0.3%)	16 (0.1%)	15 (0.1%)	1 (0.2%)	.0216	.0066
Thrombolysis therapy	528 (7.0%)	1257 (7.7%)	1190 (7.5%)	67 (10.8%)	.0028	.0532
Hospitalization						
LOS in ICU	3.55 ± 4.09	4.25 ± 5.01	4.26 ± 5.06	3.94 ± 3.70	<.0001	<.0001
LOS in General wards	6 (2, 10)	6 (2, 10)	6 (2, 10)	5 (2, 8)	.7700	.4197

Abbreviations: ACEI/ARB, angiotensin converting enzyme inhibitor/angiotensin receptor blocker; BP, blood pressure; CABG, coronary artery bypass grafting; Ccr, creatinine clearance rate; CRUSADE, can rapid risk stratification of unstable angina patients suppress adverse outcomes with early implementation of the ACC/AHA guidelines; GIB, gastrointestinal bleeding; GIP, gastrointestinal prophylaxis; GPIIb/IIIa, glycoprotein IIb/IIIa; H2RA, H2 receptor antagonist; Hb, hemoglobin; Hct, hematocrit; ICU, intensive care unit; LMWH, low molecular weight heparin; LSO, length of stay; LVEF, left ventricular ejection fraction; NSTEMI, non‐STEMI; STEMI, ST‐elevation myocardial infarction; PPIs, proton pump inhibitors; PCI, percutaneous coronary intervention; PUD, peptic ulcer disease.

^a^ PPIs vs H2RA.

^b^ No GIP vs GIP.

Among the hospitals included in the study, 66 (61.1%) had a GIP use rate of over 50%. (Figure S1A). GIP use was more frequent in provincial hospitals (74.12%) than prefectural (65.8%) and county hospitals (65.3%) (*P* < .0001). The rate of PPIs use (72.85%) was higher in provincial hospitals, while the H2RA use rate was higher in county hospitals (7.54%) (Figure S2).

### 3.2 Appropriateness of GIP use

Overall, 9086 (40.36%) patients received appropriate GIP use. The appropriate use of GIP was greater than 50% in 35 (32.4%) hospitals and only one hospital prescribed over 90% appropriate GIP to AMI patients **(**Supplementary Figure S1B**)**.

**FIGURE 2:**
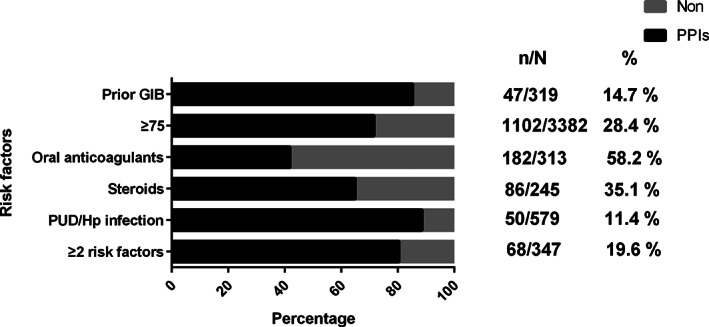
PPIs use rate in DAPT patients with high bleeding risk factors. DAPT, dual antiplatelet therapy; PPIs, proton pump inhibitors

The most common classification of inappropriate GIP use was GIP without an indication (n = 11 936, 53.3%), followed by PPIs were indicated but received alternate or no GIP (n = 1383, 6.2%). (Table S1) As for individual high GIB risk group, 58.2%, 35.1%, 28.4%, 19.6%, 14.7%, and 11.39% patients with oral anticoagulants, concurrent steroids, advanced age (≥75), 2 or more risk factors, prior GIB, PUD/*H*. *pylori* infection were not prescribed PPIs, respectively. (Figure 2).

Multivariate regression analysis showed that the independent predictors of inappropriate GIP use were GPIIb/IIIa receptor inhibitor (OR: 1.197 95% CI:1.083‐1.323, *P* = .0004) and primary PCI. (OR: 1.407 95% CI: 1.198‐1.653, *P* < .0001).

### 3.3 The impact of appropriate GIP use on clinical outcomes

Compared with inappropriate GIP, appropriate GIP use was associated with decreased GIB risk in‐hospital (OR: 0.692, 95% CI: 0.507‐0.944, *P* = .0202), while not associated with increased risk for in‐hospital NACCE (OR: 0.942, 95% CI: 0.839‐1.059, *P* = .3195) and individual endpoints. (Table 2) After 2‐year follow‐up, appropriate GIP use was not associated with increased NACCE (HR: 0.952, 95% CI: 0.857‐1.005, *P* = .3382) or GIB (HR: 0.396, 95% CI: 0.033‐4.823, *P* = .4678) risk. (Table 3).

**TABLE 2:** In‐hospital endpoints incidence and adjusted OR among DAPT patients

Clinical endpoint	Appropriate GIP use, (n = 9086)	Inappropriate GIP use, (n = 13 319)	*P* value	Adjusted OR (95% CI)	*P* value
GIB	0.8% (72)	1.0% (130)	.1473	0.692 (0.507‐0.944)	.0202
NACCE	7.6% (689)	6.2% (825)	<.0001	0.942 (0.839‐1.059)	.3195
All‐cause death	6.6% (596)	5.1% (681)	<.0001	0.978 (0.862‐1.110)	.7284
MI	0.5% (48)	0.6% (76)	.6678	0.771 (0.522‐1.139)	.1908
Stroke	0.7% (60)	0.7% (90)	.8815	0.736 (0.521‐1.040)	.0822
BARC 3/5	7.9% (44)	8.9% (58)	.5417	0.814 (0.511‐1.298)	.3880

*Note*: Variables included in the model: age, hypertension, diabetes mellitus, congestive heart failure, stroke, peripheral arterial disease, PUD/*Helicobacter pylori*, GIB; malignancy, STEMI, systolic BP, Hb, Ccr, Asprin, P2Y12 receptor, GPIIb/IIIa receptor inhibitor, oral anticoagulants, heparin/LMWH, steroids, β‐blockers, ACEI/ARB, primary PCI, emergent CABG, and thrombolysis therapy.

Abbreviations: BARC 3/5, bleeding academic research consortium 3 or 5 bleeding; GIB, gastrointestinal bleeding; MI, myocardial infarction; NACCE, net adverse cardiovascular cerebrovascular events; OR, odds ratio.

**TABLE 3:** Thirty‐day, 6‐month, and 2‐year adjusted HR of patients with appropriate and inappropriate GIP

Clinical endpoints	30‐day		6‐month		2‐year	
	Adjusted HR (95% CI)	*P* value	Adjusted HR (95% CI)	*P* value	Adjusted HR (95% CI)	*P* value
GIB	2.920 (0.235‐36.302)	.4046	0.833 (0.192‐3.608)	.8070	0.396 (0.033‐4.823)	.4678
NACCE	0.891 (0.778‐1.020)	.0950	0.936 (0.833‐1.052)	.2694	0.952 (0.857‐1.005)	.3382
All‐cause death	0.887 (0.772‐1.019)	.0902	0.941 (0.831‐1.066)	.3415	0.964 (0.862‐1.078)	.5180
MI	0.822 (0.417‐1.619)	.5705	0.927(0.611‐1.405)	.7202	0.889 (0.648‐1.219)	.4641
Stroke	0.756 (0.132‐4.310)	.7524	0.464 (0.211‐1.020)	.0560	0.641 (0.397‐1.036)	.0697
BARC 3/5	0.856 (0.211‐3.477)	.8279	1.020 (0.520‐2.004)	.9535	1.190 (0.744‐1.905)	.4676

*Note*: Variables included in the model: age, hypertension, diabetes mellitus, congestive heart failure, stroke, peripheral arterial disease, PUD/*Helicobacter pylori*, GIB, malignancy, STEMI, systolic BP, Hb, Ccr, Asprin, P2Y12 receptor, GPIIb/IIIa receptor inhibitor, oral anticoagulants, heparin/LMWH, steroids, β‐blockers, ACEI/ARB, primary PCI, emergent CABG, and thrombolysis therapy.

Abbreviations: BARC 3/5, bleeding academic research consortium 3 or 5 bleeding; GIB, gastrointestinal bleeding; HR, hazard ratio; MI, myocardial infarction; NACCE, net adverse cardiovascular and cerebrovascular events.

## 4 DISCUSSION

The main findings of the present study were as follows: (a) The GIP use rate during hospitalization in AMI patients was 68.38% and the GIP use rate was over 50% in 66 (61.1%) hospitals; (b) the overall appropriate GIP use rate was only 40.3%, inappropriate GIP use was independently associated with GPIIb/IIIa receptor inhibitor prescribing and primary PCI; and (c) Appropriate GIP use in DAPT patients was associated with decreased GIB risk during hospitalization, and was not associated with increased NACCE and GIB after the 2‐year follow‐up.

### 4.1 GIP is prevalent in China, while physicians did not follow the instructions of the guidelines

The GIP usage in the present study is higher than that reported by other registry studies, namely 22.91%, 45.83%, and 37.46% in patterns of nonadherence to DAPT in stented patients (PARIS),^7^ assessment of DAPT with drug‐eluting stents (ADAPT‐DES),^8^ PRODIGY (PROlonging Dual‐antiplatelet treatment after Grading stent‐induced Intimal hyperplasia studY trial)^17^ This was primarily because the decision to start the treatment of GIP was left to the physicians' discretion, which might be associated with a higher threshold to prescribing GIP. However, 68 (63.0%) hospitals in our study prescribed GIP to more than 50% of their AMI patients and 11 936 patients (49.7%) were overprescribed GIP indicating a lower threshold which resulted in a high GIP use rate finally.

Some studies showed that inappropriate GIP use varied from 30% to 60%,^9^, ^10^, ^18^ and overprescribing is the most frequent type of misuse.^19^ These results were consistent with those of our study, where patients with no GIP indication, but received GIP accounted for the largest proportion (83.2%) of inappropriate GIP use. After regression analysis, GPIIb/IIIa and primary PCI were independently associated with inappropriate GIP use. Although GPIIb/IIIa and primary PCI were not indications for GIP recommended by ACCF/AHA and ECS guidelines, physicians still worry that these patient groups are at a high risk of GIB, which might lead to frequent over‐prescribing. However, to the best of our knowledge, there is no large‐scale study demonstrating whether or not GPIIb/IIIa and primary PCI are independent risk factors for GIB in AMI patients.^3^ Further studies are needed to clarify the association among GPIIb/IIIa, primary PCI, and GIB.

### 4.2 The impact of appropriate GIP use on clinical outcomes

Although several studies showed that GIP, especially PPIs, can reduce GIB risk,^20^, ^21^ pharmacokinetic studies demonstrated a decrease in platelet inhibition of DAPT.^22^, ^23^ Because both PPIs and clopidogrel are prodrugs that need to be metabolized by cytochrome P450 (CYP) in liver, co‐medication would inhibit the conversion of clopidogrel to its active metabolite and altered its antiplatelet properties, which may increase the risk of ischemic events.^12^, ^24^ Therefore, routine use of GIP is not recommended by the current guideline^5^ for patients with low risk of GIB, who have much less potential to benefit from prophylactic therapy.^15^

Previous studies have focused on the effect of PPIs, but the results were inconsistent regarding their safety and effectiveness.^25^ However, few studies have investigated the impact of appropriate prophylactic therapy on clinical outcomes. In our study, those treated with appropriate GIP could benefit from prophylactic therapy, with decreased GIB risk (OR: 0.692, 95% CI: 0.507‐0.944, *P* = .0202). Furthermore, appropriate use was not associated with an increased risk for NACCE (OR: 0.942, 95% CI: 0.839‐1.059, *P* = .3195). Some clinical studies showed that inappropriate GIP use independently associated with adverse outcomes.^9^, ^18^ In our study, the results indicated that appropriate GIP use did not increase the risk for ischemic events, such as MI, stroke, and other endpoints. This was because patients who were prescribed GIP without indication were enrolled inappropriate group, while they were classified as control group against patients without GIP in previous study. These patients without indication benefited few from GIP, while the side effect of co‐medication seemed prominent which increased the patients' number of ischemic events in inappropriate group. In contrast, patients without indication who did not receive GIP were classified as appropriate group, which lower the ischemic events in this group. Therefore, in the final regression analysis, the OR was less than one for MI and stroke (without significant statistic difference). However, this emphasized the importance of compliance to the guideline.

Although, we did not find decreased GIB risk after the 2‐year follow‐up, the NACCE events were similar between the appropriate GIP use and inappropriate use groups. This indicated that appropriate GIP use only play a role in decreasing GIB risk during hospitalization. It also suggested that concomitant use of GIP, when prescribed appropriately in patients receiving DAPT, was not associated with adverse clinical outcomes in long‐term follow‐up. These results were consistent with those of another study involving PCI patients.^17^

### 4.3 Advice on health system in China

The CAMI registry, which represents a well‐supported and largest registry research, not only acts as an observational study but also serves as a resource to educate physicians and administrative personnel. Our study identified that the use of GIP is unreasonable in the treatment of AMI patients in China and patients can benefit from appropriate GIP use. This should attract the attention of physicians and administrative personnel to improve compliance with the guideline recommendations. Furthermore, a proposal should be taken to require prescribers to check an indication box when ordering PPI therapy. In addition, previous research has indicated that targeted in‐hospital educational strategy can significantly and safely reduce inappropriate PPI prescribing.^18^ Therefore, prescriber education should be provided by clinical pharmacists during hospital.^24^

## 5 LIMITATIONS

Here are some limitations in our study. First, as it is a retrospective study and over 2000 patients were excluded for missing data, the CAMI may not fully represent all AMI patients and all hospitals in China. Second, patients at lower risk for GIB with no indication for GIP who did not receive GIP (25.7%) were enrolled in the appropriate GIP group, and 6.2% patients who were indicated for PPIs, but received no drugs were included into inappropriate group. This potential confounder may have led to biased evaluation of the role of GIP (especially the exact clinical effect of PPIs). Therefore, further studies focusing on different medication‐use subgroup were warranted. Third, research showed that individual PPIs may exert different effect on clinical outcomes,^26^ further studies should identify the best PPI for AMI patients in China. In addition, GIP was not prescribed to patients routinely after discharge in our study, therefore we failed to find the association between long‐term outcomes and PPIs co‐medication.

## 6 CONCLUSION

The GIP use is prevalent in patients with AMI in China while only 40% hospitalized patients received appropriate GIP. Appropriate prophylactic therapy was associated with decreased GIB risk during hospitalization. Clinicians should pay more attention to latest guidelines and prescribe appropriate GIP to AMI patients.

## CONFLICT OF INTEREST

The authors have no potential conflict of interest.

## ETHICS STATEMENT

All procedures performed in studies involving human participants were in accordance with the ethical standards of the institutional and/or national research committee and with the 1964 Helsinki declaration and its later amendments or comparable ethical standards.

## Supporting information


**Figure S1** Frequency Distribution of Individual Hospital: Percentage of GIP use (A) and Appropriate GIP use (B)
**Figure S2** Proportion of Gastrointestinal Prophylaxis use at Different Level of Hospitals
**Table S1** GIP: Appropriate and Inappropriate in DAPT patients
